# Landscape of international event-based biosurveillance

**DOI:** 10.3134/ehtj.10.003

**Published:** 2010-02-19

**Authors:** DM Hartley, NP Nelson, R Walters, R Arthur, R Yangarber, L Madoff, JP Linge, A Mawudeku, N Collier, JS Brownstein, G Thinus, N Lightfoot

**Affiliations:** 1 Imaging Science and Information Systems Center, Georgetown University School of Medicine, Washington, DC, USA; 2 Georgetown University School of Medicine, Washington, DC, USA; 3 Pacific Northwest National Laboratory, Richland, WA, USA; 4 Centers for Disease Control and Prevention, Atlanta, GA, USA; 5 Department of Computer Science, University of Helsinki, Helsinki, Finland; 6 University of Massachusetts Medical School, Worcester, MA, USA; 7 Joint Research Centre, European Commission, Ispra, Italy; 8 Public Health Agency of Canada, Ottawa, Ontario, Canada; 9 National Institute of Informatics, Tokyo, Japan; 10 Children's Hospital Boston, Harvard Medical School, Boston, MA, USA; 11 Directorate for Public Health, Health Threats Unit, European Commission, Luxembourg, Luxembourg; 12 Health Protection Agency, London, UK

## Abstract

Event-based biosurveillance is a scientific discipline in which diverse sources of data, many of which are available from the Internet, are characterized prospectively to provide information on infectious disease events. Biosurveillance complements traditional public health surveillance to provide both early warning of infectious disease events and situational awareness. The Global Health Security Action Group of the Global Health Security Initiative is developing a biosurveillance capability that integrates and leverages component systems from member nations. This work discusses these biosurveillance systems and identifies needed future studies.

## Introduction

Far from being conquered by public health, vaccines, and antibiotics, infectious diseases continue to threaten human- kind globally. There is a rich contemporary literature regarding the burden of endemic disease and epidemics of age-old threats, the emergence of newly discovered pathogens, drug resistance and the phenomenon of reemerging microbial threats.^1–3^ In addition, biological terrorism remains a clear and present danger.^4^ Beyond the personal impact on individuals suffering from infection, disease has societal impact: it can destabilize social institutions, populations, economies, and governments. For this reason, infectious disease is both a national and an international security issue.^5, 6^

The prevention and control of infectious diseases is therefore of extreme importance. World mobility rose significantly throughout the twentieth century and it continues to increase. Relative to past decades, people are traveling more, and travel times are dramatically shorter; at present it is possible to circumnavigate the globe in 36 h through regularly scheduled commercial flights.^2^ More people, living species, and agricultural commodities are crossing borders than ever before, increasing the likelihood that pathogens circulating in one area will be translocated to another area. One of the consequences of such global mobility is that disease prevention in any one area often depends on the effectiveness of surveillance, communication, and response control in other areas.^7^

Early warning of outbreaks may enable targeted quick intervention and control activities to take place. This was a motivation behind the 2005 revisions of the International Health Regulations (IHR).^8, 9^ The IHR-2005 provide an international legal framework for the early detection and reporting of, and response to, outbreaks of infectious disease. WHO member nations are obligated to develop and maintain surveillance, reporting, notification, verification, and response capabilities. Any nation with knowledge of a disease outbreak of international concern is obligated to report it to the WHO within 24 h. The IHR-2005 are designed to ensure timely recognition of disease outbreaks of international public health significance and to promote effective containment before they spread.

Historically, many epidemics have been reported through informal networks of health workers. Such networks should be timely, to assist in rapid detection, and sensitive, to detect potentially important outbreaks. As such, they may differ from traditional public health surveillance alluded to in the IHR, which often rely on classical epidemiologic studies or clinical or laboratory data, the availability of which often lag the events they describe by days or months. This approach can also be less specific than traditional public health surveillance, although such trade-offs may be appropriate for a network designed to provide early warning.

Surveillance has been enhanced by the development of several novel approaches complementing traditional methods.^10^ Event-based biosurveillance is a new scientific discipline that uses information from the Internet whereby diverse streams of data are characterized prospectively to provide information on events affecting human health.^11^ Indicator-based systems rely on routine collection of structured data such as syndromic surveillance and clinical activity monitoring, whereas these new event-based systems use unstructured data from media and other sources to detect anomalies that may indicate an emerging threat.^11^ The potential of biosurveillance to contribute to global early warning of infectious disease and related threats, including chemical, biological, radiological, and nuclear (CBRN) agents, is becoming recognized.^12^ Researchers have developed prototype Internet-based systems to monitor and track the emergence of infectious disease and to evaluate the degree to which biosurveillance can provide early warning of outbreaks.^13^

Founded in 2001, the Global Health Security Initiative (GHSI) is an informal international partnership to strengthen health preparedness and response globally to CBRN terrorism threats and pandemic influenza.^14^ Partners include Canada, European Union, France, Germany, Italy, Japan, Mexico, the UK and the United States with the WHO holding observer status. A Global Health Security Action Group (GHSAG) of senior officials from partner nations has been established by the GHSI to develop and implement concrete actions to improve global health security. The GHSI/GHSAG has established a number of working groups on areas such as smallpox, risk management and communication, chemical incidents, and pandemic influenza.

A GHSAG senior official meeting (in Ottawa, Canada, in June 2007) identified CBRN early warning as an area with great potential to support the efforts of GHSAG. A meeting of the Risk Management and Communications Working Group (RMCWG; in Luxembourg, February, 2008) focused on identifying, within the context of CBRN hazards and risks, the capacities and input needs of existing IT systems working currently in the early detection of public health threats.^15^ The RMCWG is currently making preliminary assessments of the opportunities, with a focus on bioterrorism and diseases threatening public health. A follow-up meeting in Ispra, Italy explored in detail the tasks of each proposed work package in preparation for the Ninth Ministerial Meeting of the GHSI in Brussels, Belgium in early December 2008. In 2007–2008, the GHSAG made progress addressing key risks to global health security. This was accomplished through a variety of technical, scientific and policy networks and initiatives, and stemmed from collective efforts and approaches in areas such as prevention, research, preparedness, and response. In combination, the GHSAG event-based surveillance systems, which use the media as the primary source of information, form a unique part of the landscape of international biosurveillance.

## Methods

This review covers GHSAG-member biosurveillance systems, which constitute a major (although incomplete) fraction of similar capabilities available to the public health community at present. We elicited basic information from the respective system investigators to compare and contrast system capabilities and to illustrate the complementarities of the different approaches to event-based biosurveillance. Each biosurveillance system described in this study has been approved by institutional review board or corresponding authority at the respective institutions housing the systems.

## Systems

Several systems originating from GHSAG member nations with a focus on biosurveillance or situation awareness are known at present and are described in this section. Table 1 provides a brief comparison of system traits and capabilities. The systems are listed alphabetically; no ranking should be inferred from the order of presentation.

**Table 1:** Comparison of system traits and capabilities

*System name*	*Argus*	*BioCaster*	*GPHIN*	*HEDIS*
Sponsoring agency	US Government	National Institute of Informatics in Japan	Canadian government	European Commission
Access policy	Limited	Open to public	Fee-based	Restricted
Posts per day (Approx.)	120–170	90	2000	NA
Staff	45	5	9 daytime	5 Ispra
			6 nighttime	3 Luxembourg
Ceographical coverage	Worldwide, not US	Asia-Pacific región	Worldwide	Worldwide
Languages	40	3	9	NA
Update frequency	Every 1–15 min	Every hour	Every 20 min	News: continuous Documents: when uploaded
Covered topics	Human, animal, plant diseases, enviro-climatic indicators	Human, animal infectious diseases	Human, animal, plant diseases; chem/rad events; ‘unsafe producís’ and natural disasters	Health threats
Information disseminated	Watch-board and alerts	Watch-board	Watch-board; approx. 1–4 ‘alert’ emails delivered to subscribers daily	Watch-board
Staging or scoring system	Yes, 7-stage social disruption scale at the event level; low, médium, high level of importance at the country level	No	‘Relevancy Scoring’ system and IHR related decisión tree is applied that provide email'alerts'	No
Direct indicators and warning	Public health	Public health	Public health	Public health
Indirect indicators and warning	Public health response, meteorological data, other government reaction, business/ organization changes, other social behavior	No	Public health response, other government reaction, meteorological data	No
*System name*	*HealthMap*	*MedISys*	*ProMED-mail*	*PULS*

Sponsoring agency	Children's Hospital Boston, Harvard University, soft monies	European Commission	ISID, soft monies	University of Helsinki and European Commission's Joint Research
Access policy	Open to public	Open to public	Open to public	Center Public and restricted views
Posts per day (Approx.)	30–300	Not applicable	7	300
Staff	6	20 (for both MedISys and Europe Media Monitor)	30	7
Ceographical coverage	Worldwide	Worldwide	Worldwide	Worldwide
Languages	7	26	4	2
Update frequency	Every hour	Every 10 min	Variable	Every 20 min
Covered topics	Human, animal, and plant diseases	Human and animal diseases, and very loosely defined'health' topics	Human, animal, plant diseases	Human, animal, plant diseases
Information disseminated	Watch-board, automated email alerts, RSS, mobile phone, Twitter	Watch-board; email ‘alerts’ autodelivered to subscribers	Watch-board	Spreadsheet-like tables
Staging or scoring system	An 11-staged ‘Heat Index’ system	Alerts at low, médium, and high levels are assigned to reports	No	Relevance scoring: importance to user (1–5); system confidence score
Direct indicators and warning	Public health, clinical, laboratory	Public health	Public health, clinical, laboratory, veterinary	Public health
Indirect indicators and warning	Public health, environmental disasters, conflicts	No	Public health	No

Abbreviations: ISID, International Society for Infectious Diseases; NA, Not applicable.

### Argus

Project Argus is a prototype biosurveillance system designed to detect and track biological events that may threaten human, plant, and animal health globally.^16^ The approach is based on monitoring social disruption evident in local, native-language media reports around the world. Argus uses analysts speaking approximately 40 languages to monitor a large number of media sources including traditional print and electronic media, Internet-based newsletters, and blogs. It alerts users to events that may signal the initiation of outbreaks and shows trajectories of events that may require additional investigation. Bayesian analysis tools are used for article selection and alerting.

### BioCaster

BioCaster (http://www.biocaster.org) is an experimental system for global health surveillance under development at the National Institute of Informatics in Japan, and is a collaborative research project among five institutes in three countries.^17^ The system is fully automated using Really Simple Syndication (RSS) feeds from more than 1700 sources with no human analysts. Human analysis is assumed to take place downstream by the recipients of its output. BioCaster focuses on the Asia-Pacific region, posting approximately 90 articles per day in three languages (English, Japanese, and Vietnamese) with plans for expansion to Thai, Chinese, and other regional languages. Article capture and dissemination is carried out every hour. Until recently, the primary sources are Google News, Yahoo! News, European Media Monitor, but the system is now expanding to take on sources from a commercial news aggregation company greatly increasing its coverage. BioCaster produces an ontology^18^ in eight languages (Chinese, English, French, Japanese, Korean, Spanish, Thai, and Vietnamese) that is openly available and is the basis for the Global Health Monitor,^19^ an open access Web portal for showing maps and graphs of health events to users. The ontology covers approximately 117 infectious diseases of humans and animals as well as six syndromes. Future objectives include extending language and health threat coverage.

### Global Public Health Intelligence Network

Global Public Health Intelligence Network (GPHIN; http://www.phac-aspc.gc.ca/gphin/index-eng.php) is the principal system used by WHO Alert and Response Operations for monitoring media articles.^20^ It was established in 1997 and is managed by the Public Health Agency of Canada's Centre for Emergency Preparedness and Response. It covers nine languages, Chinese Simplified and Traditional, Portuguese, Spanish, French, Russian, Arabic, English, and Farsi. It provides 24-h operation seven days per week coverage and applies human-based triage of information. Outputs are presented to GPHIN's user community (WHO, public health, intelligence, and law enforcement officials) as raw, machineselected, and translated articles. Machine selection of articles is based on keywords corresponding to the IHR. The online sources of GPHIN are drawn from sources available from Factiva and Al Bawaba, supplemented by automated and manual Web crawling performed by GPHIN analysts. Future objectives of GPHIN include integration of verification networks, addition of other news sources, additional languages, use of geographic and data visualization, and incorporation of audio/visual feeds.

### Health Emergency Disease Information System

Health Emergency Disease Information System (HEDIS; http://hedis.jrc.it/), based in Italy, is a situation awareness tool developed by the European Commission and aimed primarily at crisis management. It supports the Health and Consumer Protection Directorate Genera (DG SANCO) and public health authorities in member states. There are approximately 300 users in Europe who use the system as an interorganizational information-sharing platform to assist customers in dealing with an identified health threat. Users are member states responsible for communicable disease and CBRN threats and risk communications. Although HEDIS is not used for routine biosurveillance, in times of crisis it integrates biosurveillance information from many of the systems described in this study and makes it available to public health authorities.

### HealthMap

HealthMap (http://www.healthmap.org/about.php) is a multilingual, real-time disease outbreak detection, tracking, and visualization system.^21, 22^ Launched in fall 2006, the Web site collects more than 300 reports per day in English, Spanish, French, Russian, Portuguese, Arabic, and Chinese, from both general news media and public health sources around the world. Sources include Google News (in all seven languages) as well as other online news aggregators and informal sources, along with Program for Monitoring Emerging Diseases (ProMED) and WHO. The system also allows for user-provided reporting through submission of URLs. Updated hourly, the system filters reports to determine relevance, disease, location, and duplication clustering by means of a series of automated text processing algorithms. Relevant reports are then aggregated and shown in a freely available dashboard where users can tailor the view according to date, disease, location, and source. Although the system is fully automated, dedicated human analysts along with collaborators at US Centers for Disease Control and Prevention, the UK Health Protection Agency (HPA), WHO, and ProMED examine reports each day to verify the accuracy of the system output. To date (as of 1 March 2009), the system has collected more than 150,000 reports, covering 191 disease categories and more than 200 countries and autonomous territories. With 1000–150,000 visits per day (with top visitors from government, academic, and public health agencies), HealthMap provides an overview of real- time information on emerging infectious diseases and has particular interest for public health officials and international travelers. Future system development is particularly focused on participatory surveillance, where users can contribute, edit, and comment on disease intelligence as part of an online social network.

### Medical Information System

Medical Information System (MedISys; http://medusa.jrc.it/medisys/aboutMediSys.html) is a fully automatic 24/7 public health surveillance system run and maintained by the Joint Research Centre (JRC) of the European Commission. The developer team collaborates with the Health Threats Unit at the European Union Directorate General for Health and Consumer Affairs (DG SANCO) and the University of Helsinki (Pattern-based Understanding and Learning System, PULS system). MedISys covers infectious human and animal diseases and CBRN threats reported in open-source news media. Approximately 90,000 articles from 5000 news pages in 45 languages are screened. Currently, 26 languages are available through the Web portal, but news in all 45 languages is processed in predefined categories. Users can access world maps in which event locations are highlighted, graphs showing aggregated news counts by disease-location for an alerting category, graphs showing the most significant disease-location pairs for the last 24 h, alerting statistics for regions of the world, filtering of news according to language, disease, or location, and filtering by orthogonal categories such as ‘outbreaks’, ‘treatment’, ‘legislation’, and showing specific entities within the news article such as persons, organizations, and search words. MedISys provides daily automated e-mail alerts to subscribers and offers a tool called Rapid News Service in which users can manually select articles into predefined categories, comment on them, create formatted newsletters, and distribute these to user-defined groups. MedISys became online in August 2004 and is one of several JRC-developed media-monitoring applications that process news gathered by the Europe Media Monitor (EMM, online since 2002). Therefore, future developments on EMM will also benefit MedISys.^23–25^

### Program for Monitoring Emerging Diseases

ProMED (http://www.promedmail.org) was established in 1994 and currently operates as a program of the International Society for Infectious Diseases with contributing corporate, foundation, and individual donor support.^26, 27^ It is an unautomated, human-driven process, where more than 40,000 freely subscribed members in more than 160 countries submit reports of disease. The majority of these reports are media articles. Other sources include local observers, official reports, and others. All reporting is screened by subject matter experts before posting (approximately seven reports issued per day). A total of 50,000 reports have been posted since project inception in the mid 1990s (10,000 of which are veterinary disease reports). ProMED has approximately 30 staff member subject matter experts, five regional programs, and staff in 15 countries. Regional programs of ProMED include Latin America, the Mekong Basin, the East Africa Integrated Disease Surveillance Network, and ProMED-RUS (former Soviet Union). ProMED-mail is available in English, Spanish, Portuguese, and Russian languages. Future objectives include French language reporting.

### Pattern-based Understanding and Learning System

PULS (http://puls.cs.helsinki.fi/medical/) is a project at the University of Helsinki, in collaboration with the European Commission's MedISys, and the European Centre for Disease Prevention and Control (ECDC). PULS traces its origins to the IFE-BIO Project, which aimed to analyze events reported in ProMED-mail.^28^ As ProMED-mail, PULS tracks human, animal, and plant diseases, currently covering more than 1500 base terms, with 2500 variants. The focus in PULS is on the analysis of news texts for information extraction, aggregation, and visualization. PULS is fully automated with no human intervention. It uses MedISys as its main source, and uses natural language processing methods for analyzing the news stream to build a database of facts about the epidemiological events. The output of PULS is a spreadsheet- like view of the fact base, which is updated every 20 min. The base is also Google Earth-enabled. Linguistic coverage is primarily English, with a recent introduction of French language analysis. The PULS average daily extraction rate varies from 300 entries during ‘normal’ periods to more than 1000 per day during times of heightened reporting, totaling about 300,000 entries to date. Future objectives include stronger multilingual support (with the addition of Spanish, Russian, and Chinese), trend analysis, and data visualization.

## Discussion

Event-based biosurveillance possesses strengths and limitations that make it complementary to other experimental as well as traditional public health surveillance. Such systems may not always be timely, they may have limited specificity, and baseline thresholds for indicator detection may be difficult to quantify. Although the systems described above are representative of the rapidly changing state of the art in event-based biosurveillance, important technological and methodological challenges remain.^29^ Prominent challenges include interoperability, interface customizability, scalability, and event traceability. Integration of geospatial visualization, event mapping, modeling and trending tools are important for establishing metrics and baselines necessary for data interpretation and analysis. In addition, expansion of the current biosurveillance capability by incorporation of emerging media such as video, audio, images, blogs, social networking sites, SMS (short message service) and others may be important.

Although some qualitative aspects of recognizing important public health threats using event-based surveillance are evident, the value of diverse data sources must be quantified. Given the diversity and richness of the Internet, and the availability of data and information from other sources (for example, traditional public health, syndromic, and laboratory surveillance) of varying degrees of confidence and geographic coverage, how to quantify the payoff of including different sources in biosurveillance systems is unclear. Quantifying variation in source reporting standards as well as catchment (that is, the regions from which a source collects data) and target population will be important for understanding the validity of biosurveillance system output. Metrics must be defined, and these metrics need to be generalizable across systems using different data and different approaches to analysis.

Standard guidelines for evaluating public health surveillance systems may not be wholly appropriate for evaluating event-based biosurveillance systems.^30^ Techniques for evaluating system performance are needed and standardized metrics quantifying the performance of distinct biosurveillance systems must be developed. Such metrics are also needed if end users are to be able to understand the performance of a given system, or an aggregation of systems. Similarly, analytic methods for assessing and quantifying the value added by biosurveillance to other approaches to surveillance and situational awareness must be developed.

Efficient and meaningful ways of communicating complex biosurveillance data must be identified. Because they are tailored to meet the needs of the specific user communities, current systems show and present the results of biosurveillance differently. How to best present results to the broader user community, which includes researchers as well as public health workers and decision makers, is unclear. Many unknowns remain, including identifying the most appropriate interactive visual interfaces; best practices regarding techniques for synthesizing biosurveillance data visually; and how to present dynamic, ambiguous, and potentially conflicting information to consumers of biosurveillance.

Real-time situational awareness of emerging biological threats is needed in today's dynamic world. However, if such an approach to public health response is to be viable, a capability must exist to detect evidence of outbreak activity at the earliest stages and monitor related information as it evolves. We are unaware of published studies investigating the timeliness of event-based biosurveillance using Internet sources relative to traditional approaches to public health surveillance. To maximize the likelihood of early detection, such a capability should be composed of discrete components acting in concert. At one end of the alerting spectrum, biosurveillance systems that provide indications and warning (I&W) of potential infectious disease events are needed. These I&W components would provide the first tip of a potential event or risk of a future event. Necessarily, information provided by systems at this end of the spectrum would have limited confidence and their output would need to be refined and better characterized by other components in the alerting spectrum. Toward the middle of the spectrum would be systems that more directly measure infectious disease activity, for example, syndromic surveillance systems. At the opposite end of the spectrum would be traditional formal clinical and laboratory-based public health surveillance.

At the biosurveillance end of the spectrum, there is considerable variation in system capability, data analyzed, and products disseminated, pointing to the need for integration. A meeting of GHSAG participants (Luxembourg, 2008) highlighted the need for ‘cooperation at all levels, between systems, between systems and users, and users amongst themselves. Such cooperation should be considered at the level of the collection of data, at the level of data analysis of the data available and the subsequent sharing of the relevant information through a common restricted platform.’^31^

Although such a capability does not yet exist, similarities and differences among the systems described above suggest that combining these approaches into a single system can provide a powerful biosurveillance resource. The GHSAG is developing such a prototype biosurveillance ‘system of systems’; it is anticipated that, with appropriate communication and data sharing protocols, technical barriers to integrating existing global and regional biosurveillance systems can be overcome. It is possible partially because each of the individual systems examined here has different missions and approaches, and complement one another. This complementarity will be shown in the GHSAG pilot integration project.
